# The Experiences of Nurses as First Responders to Disaster: A Qualitative Study

**DOI:** 10.1111/nhs.70084

**Published:** 2025-03-30

**Authors:** Cafer Açık, Tuğba Yeşilyurt Sevim

**Affiliations:** ^1^ Istanbul Europe Provincial Ambulance Service 112 Emergency Health Services İstanbul Turkey; ^2^ Nursing Department Istinye University Faculty of Health Sciences Istanbul Turkey

**Keywords:** disaster, disaster nursing, earthquake, nurse, patient care

## Abstract

This study aimed to explore the experiences of nurses who were the first to respond to the disaster, using a descriptive qualitative design. The sample consisted of 22 nurses who were nurses in the earthquake‐affected provinces of Turkey or who were on assignment in the region after the disaster. Data were collected through individual in‐depth interviews and analyzed using content analysis. The findings are categorized into four main themes: the assignment process, practical challenges, psychosocial challenges, and positive experiences. Under the assignment process, sub‐themes of voluntary and non‐voluntary assignment are identified. Practical challenges include ineffective crisis management, communication and coordination issues, lack of medical materials and equipment, difficulty working in tents, triage challenges, lack of personal hygiene, and nurses' lack of knowledge and skills. Psychosocial challenges encompass emotional burden, feelings of inadequacy, safety concerns, and the challenges of post‐disaster recovery. Finally, positive experiences are reflected in the sub‐themes of professional satisfaction and team support. The findings emphasize the need for disaster preparedness in health systems to enhance nurses' response capabilities and recommend that policymakers develop effective disaster management strategies.

**Summary**

- Nurses play a critical role in disaster planning, response, and recovery.
- Nurses face various physical, psychosocial, and organizational challenges in responding to disasters.
- Disaster preparedness is of great importance in health systems for nurses to be able to respond effectively to disasters and for the strengthening of their role.

## 1 Introduction

Disasters and emergencies are emerging as important public health issues globally (İytemür and Yeşil 2020). Among natural disasters, earthquakes are among those that have the greatest impact on societies (Altun 2019). Turkey, which is located in an earthquake‐prone zone, has experienced many earthquake disasters throughout its history (Şen 2023). On 6 February 2023, the 7.7 and 7.6 magnitude earthquakes, centered in the Kahramanmaras province, affected more than 13 million people across 11 provinces in Türkiye. These earthquakes caused a total of 50 096 deaths and 107 204 injuries (AFAD 2023). The earthquake, which occurred on the East Anatolian Fault and affected 11 provinces, was recorded as the most devastating earthquake of the last century (Şen 2023).

Although disasters and emergencies affect all sectors, the health sector is one of the most affected. The demand for health services increases significantly after disasters (Doğan et al. 2024; Erdoğan 2018). Nurses, as the largest group of health professionals, play a critical role during the post‐disaster period. Their competence in disaster response is crucial for mitigating the adverse health outcomes experienced by those affected (Brewer et al. 2020; Doğan et al. 2024).

### 1.1 Background

The provision of health services is one of the most demanded services by society during disasters and is expected to be delivered with high quality (Erdoğan 2018). Nurses, who make up a large part of the healthcare system, play a vital role in ensuring safe and effective disaster recovery and response. Furthermore, in all phases of disaster management, nurses can play a vital role in the reduction of disaster risks, complications, and loss of life through education, management, and leadership, counseling, support, guidance, and research (Farokhzadian et al. 2024). Therefore, nurses are considered to be the most important health professionals who are constantly involved in disasters, which imposes important responsibilities on them in disasters and emergencies (Erdoğan 2018; Xue et al. 2020). This responsibility requires nurses to be professionally prepared for disaster management and to have disaster care planning and implementation skills, which are critical for safe and effective rescue and response (Li et al. 2015). The International Council of Nurses (ICN) emphasizes that disaster preparedness and response should be part of every nurse's knowledge and skills (ICN 2022; Xue et al. 2020).

Nurses play a critical role in disaster planning, response, and recovery. Although there are many quantitative studies on the role of nurses in disaster response, these studies highlight the basic elements of the role of nurses in disaster response, but do not provide detailed insights into their experiences. Quantitative studies do not fully assess nurses' perspectives, intentions and role perceptions in disaster response. In addition, they do not adequately reflect the impact of environmental, individual, legal, and organizational factors on nurses' ability to respond to disasters (Geum 2017). Although there is a need to develop disaster nursing education to effectively prepare nurses to work following a disaster, this should be based on evidence derived from the reflections of nurses who have experienced responding to disasters. Therefore, by exploring nurses' perspectives and experiences after responding to a natural disaster, this study will make a significant contribution to identifying areas for improvement in nurses' disaster response processes and providing evidence for disaster management policy.

## 2 Methods

### 2.1 Aim

The study was conducted to determine the experiences of nurses who were the first to respond to the disaster about their experiences.

### 2.2 Design

A descriptive phenomenological design was used in this study (Giorgi 1997). This approach helps clarify the nature of a phenomenon by exploring the experiences of research participants from various perspectives and uncovering the meanings embedded in those experiences (Maxwell 2013). The Consolidated Criteria for Reporting Qualitative Research (COREQ) checklist guides the reporting of this study (Tong et al. 2007).

### 2.3 Research Team

The research team consisted of two professional academic researchers (male, female) (CA, TYS). Both worked as clinical nurses and were trained in qualitative research. The pilot interviews were supervised by TYS. There was no prior interaction between the researchers and the volunteers.

### 2.4 Sample and Settings

The research was conducted between November 2023 and March 2024 through online interviews with participants who agreed to participate in the study.

Due to the characteristic of using more than one sample simultaneously in qualitative research (Yıldırım and Şimşek 2016), two sampling strategies, maximum variation sampling and snowball sampling, which are among the purposive sampling methods, were used because they provide the opportunity to reflect the diversity of people who can participate in the study to the greatest extent. Inclusion criteria were working as a nurse in the region during the disaster or being assigned to the region after the disaster for intervention and volunteering to participate in the study. The first step was to contact nurses who worked in the 11 provinces hit by the earthquake during the disaster or who were assigned to the earthquake zone for disaster relief and could direct the other participants who could be interviewed in the study. Through these nurses, the names and contact information of nurses who varied in terms of gender, age, length of experience, and province of service were obtained to ensure maximum diversity, and the sample was expanded, and nurses who agreed to participate in the study were included in the sample group. Nine nurses who were contacted refused to participate in the study, citing their busy schedules. Data saturation (Sandelowski 1995) was continuously assessed, and the study was stopped at 22 samples when no new data were provided.

### 2.5 Measurements

In the study, data were collected using a semi‐structured interview form developed by the researcher based on the literature (Farokhzadian et al. 2024; Xue et al. 2020; Yang et al. 2010), which consisted of open‐ended questions designed to explore the nurses' disaster response experiences in more detail (Table 1).

**TABLE 1:** Semi‐structured interview questions.

Can you share your experience of participating in disaster response activities?
What do you think about disaster management and disaster nursing?
How did you feel when you heard about the earthquake/when the earthquake happened?
What was your experience of caring for patients affected by the earthquake?
What kind of situations did you encounter during your interventions in the earthquake zone and how did you copy with them?
Based on your experience, what suggestions do you have to improve the quality of disaster nursing and health services?

### 2.6 Data Collection

The first author contacted participants by telephone to provide details of the study and an invitation to participate. For those who agreed to participate, interview dates were arranged according to their availability. Prior to data collection, two pilot interviews were conducted to assess the clarity and comprehensiveness of the format and semi‐structured questions. Data from the pilot interviews were not included in the final study.

The first author conducted individual, in‐depth online interviews (via video call) using the semi‐structured interview form. Prior to the interview, the researcher provided the nurses with the informed consent form, which included the necessary explanations about the purpose of the study, the recording of audio and video, the benefits of the study, and the confidentiality of their identity. The researcher then confirmed these explanations on the recording and obtained verbal consent. Interviews with nurses who consented to audio‐video recording were recorded by the researcher. The interviews were concluded by asking the participants if they had any additional thoughts. The interviews were conducted between October 2023 and December 2023 and lasted between 28 min and 1 h and 48 min.

### 2.7 Ethical Considerations

The research was approved by an ethics committee (Decision number and date: 23‐243/September 22, 2023). Informed consent was obtained from the participants. The names of the participants were kept confidential, and the results were reported by assigning numbers to each participant.

### 2.8 Data Analysis

The data were analyzed using an inductive content analysis technique through the following steps (Graneheim and Lundman 2004; Vaismoradi et al. 2013). Content analysis is a systematic coding and categorizing approach used to describe the concepts and relationships that explain the data obtained from interviews with participants (Elo and Kyngäs 2008; Vaismoradi et al. 2013). It is particularly well suited for analyzing important, multifaceted, and sensitive nursing phenomena (Vaismoradi et al. 2013). In inductive content analysis, the process moves from the specific to the general, meaning that specific topics are first observed and then combined into broader statements without imposing preconceived categories (Elo and Kyngäs 2008; Vaismoradi et al. 2013). In the first stage of qualitative data analysis, the interviews recorded with audio recorders were transcribed into written text by the researcher using a computer. During this process, the audio recordings were listened to carefully, and the participants' statements were transcribed verbatim without any changes. The written transcripts were read multiple times by both researchers. Each researcher independently examined the transcripts to identify the conceptual meaning of each statement and then developed explanatory codes that would give meaning to these statements as a whole. After identifying recurring or unifying concepts in the text, the researchers classified data with similar interpretations under the same codes. The codes were then merged and categorized. In the data analysis process, MAXQDA 24 qualitative data analysis software was used to organize the data. During the abstraction process of the study, the researchers re‐evaluated and compared the themes and sub‐themes, reaching a consensus on the final thematic structure.

### 2.9 Rigor and Trustworthiness

The trustworthiness of this research was ensured by using Guba's four criteria: credibility, transferability, dependability, and confirmability (Guba 1981). To enhance the credibility of the study, the researchers employed multiple strategies. First, pilot interviews were conducted to assess the comprehensiveness and clarity of the data collection tool, ensuring that the questions were understandable for the participants. All interviews were audio‐recorded and transcribed verbatim. In reporting the data, sample quotes were taken directly from the interviewees and presented as faithfully as possible, without added commentary. Member checking was also used, where transcripts were returned to participants for review and feedback, allowing them to confirm or clarify the accuracy of their responses. This process helped ensure that the findings accurately reflected the participants' true experiences. The researchers independently analyzed the data and reached consensus on the final themes.

For transferability, purposive sampling was used to ensure a diverse range of participants, with the findings supported by direct quotes and illustrative examples. To enhance dependability, data collection was conducted by a single researcher to ensure consistency across the interviews, and transcriptions were completed on the same day. For confirmability, the researcher documented participants' responses, behaviors, and observations during the interviews to minimize bias in the data collection process. Finally, all study materials, including transcripts and coding documents, were securely stored to ensure transparency and enable future verification, allowing for the validation of the study's findings.

## 3 Results

It was found that the duration of nurses' professional experience varied from 1 to 17 years (average = 5.73 ± 2.11) and most of their work area before disaster was in surgical and emergency services. The majority had baccalaureate degrees (81.9%) (Table 2).

**TABLE 2:** Descriptive characteristics of nurses (*N* = 22).

	*N*	%
Gender		
Male	11	50.0
Female	11	50.0
Educational background		
High school	1	4.5
Baccalaureate degree	18	81.9
Graduate degree (MSc or PhD)	3	13.6
Work area before disaster		
Emergency service	5	22.7
Surgical service	7	31.8
Intensive care unit	4	18.2
Other	6	27.3
Province of assignment		
Hatay	12	54.8
Adıyaman	2	9.0
Gaziantep	1	4.5
Kahramanmaraş	5	22.7
Adana	1	4.5
Malatya	1	4.5
Duration of professional experience (years)		
≤ 5	9	41
5–10	11	50
≥ 10	2	9

**FIGURE 1:**
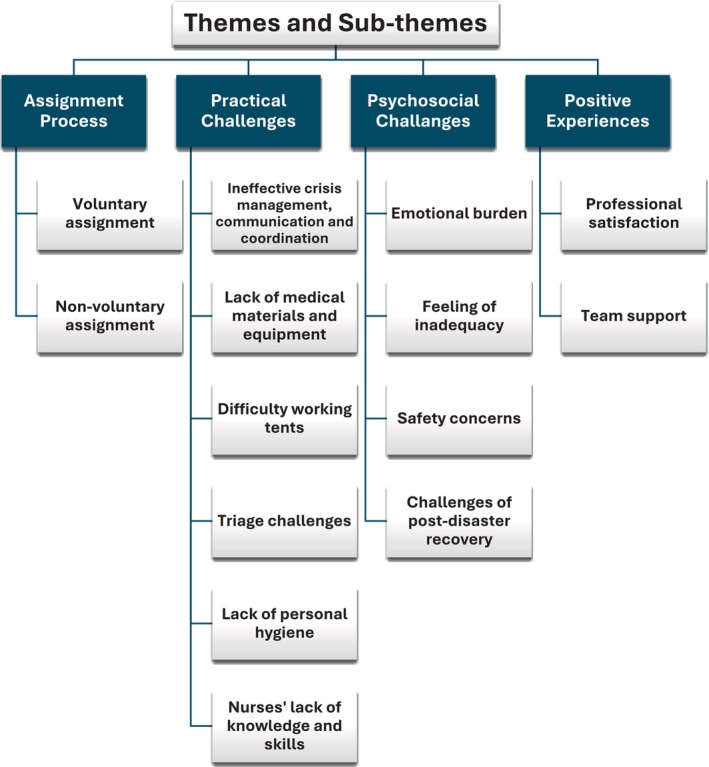
Themes and sub‐themes.

The data were organized under four main themes and 14 subthemes (Figure 1). The four main themes are assignment process, practical challenges, psychosocial challenges, and positive experiences. Each theme is presented by adding selected quotes (translated from Turkish) from the nurses' experiences.

### 3.1 Main Theme 1: Assignment Process

Nurses said they went to the earthquake zone through their organizations. Due to the scale of the disaster, which affected 10 provinces, there was a great need for health services. The Ministry of Health assigned nurses from different provinces to meet the emergency health needs in the affected areas. Initially, priority was given to nurses who volunteered. However, due to the magnitude of the healthcare needs, it was not possible to manage the process with volunteers alone, and assignments were subsequently made without regard to the volunteer criterion. Priority was given to nurses working in critical areas such as intensive care, emergency services, dialysis and operating rooms. Under the theme of the assignment process, the sub‐themes of ‘voluntary assignment’ and ‘non‐voluntary assignment’ were discussed.

#### 3.1.1 Voluntary Assignment

The initial assignment made by the Ministry of Health was based on voluntarism. Nurses who volunteered for the assignment highlighted a strong sense of personal responsibility and commitment to their role as healthcare professionals. Many nurses felt an intrinsic duty to help, particularly during such a critical time.

…They announced the provinces where we would be sent and asked if any nurses wanted to go voluntarily. Those who volunteered went first… (16).

…I thought, if I don't work today, when will I work as a health worker… I felt very responsible and we went straight to the region… (5).

…I said they needed me and I volunteered… (18).

…there was a message from the management (hospital administrators) asking if anyone wanted to go as a volunteer. As a priority, they wanted more nurses from intensive care, emergency, dialysis, operating rooms… (17).

#### 3.1.2 Non‐Voluntary Assignment

Participants reported that non‐voluntary assignments were made after finalizing the voluntary lists. Moreover, nurses living and working in the disaster area, although directly affected by the disaster, were also called upon by their institutions to ensure the continuity of healthcare services. Some participants stated that non‐voluntary assignments created additional challenges, particularly for those affected by disasters.

…After the volunteer lists were completed, mandatory assignments began. The needs in the region were immense, which made the mandatory assignment inevitable… (5).

…I was also a disaster victim. However, immediately after the earthquake, organization I worked for mandated everyone to return to work at the hospital. Working under these conditions was very difficult for me… (1).

### 3.2 Main Theme 2: Practical Challenges

In analyzing the findings under the heading of ‘practical challenges’, they were grouped under six sub‐themes: ‘ineffective crisis management, communication and coordination’, ‘lack of medical materials and equipment’, ‘difficulty working in a tent’, ‘triage challenge’, ‘lack of personal hygiene’ and ‘nurses’ lack of knowledge and skills'.

#### 3.2.1 Ineffective Crisis Management, Communication and Coordination

Nurses expressed problems in coordinating and communicating during the crisis management.

…I didn't even know where to go. They said we were going on a mission and we went. I even went as a team leader. The team asked me where we were going, but I didn't know where we were going… (13).

…it was completely like a battlefield. For example, nobody directed us, we couldn't find a manager… (6).

…sometimes I even had the feeling that I was superfluous, so that if there were an unnecessary number of people there, no one could intervene… (22).

…The communication between the management tent and field staff was disconnected. The radios stopped working beyond a certain range, making coordination between the field and management difficult … (21).

…The communication breakdown complicated both logistical processes and patient transfers. Even the placement of incoming containers was chaotic… (13).

#### 3.2.2 Lack of Medical Materials and Equipment

Nurses reported that they provide services with limited resources. In particular, they stated that the lack of medical materials and equipment causes them great difficulties. They emphasized that the lack of critical equipment, such as respirators, hinders the provision of basic health services, and that delays and complexities in the supply of materials have a negative impact on the effectiveness of health services in times of crisis.

Because we were in a tent, we had very little medical equipment. We had no respirator and so on. We tried to do as much as we could… We used as much material as we could get from the hospital. When that ran out, we had to go into the building, even if it was damaged, to get material. Because our material supply came very late and was not delivered to us… (2).

#### 3.2.3 Difficulty Working in Tents

Nurses reported that the cramped and overcrowded tents made working with patients difficult and prevented effective care.

It was really difficult to do interventions in the tent. Because in the hospital environment you know more or less what is where. But in the tent it is chaos. We could not do most of the care in the tent environment. Because it was too crowded… (13).

#### 3.2.4 Triage Challenges

Nurses highlighted the challenge of triage in the field, in tents, or in hospital.

…It was very difficult to organise the order of the patients and intervene according to their urgency. The tent was overcrowded, everything was chaotic… Poor organisation made triage very challenging… (13).

…In the tent, we performed quick triage and prioritised the most urgent patients. However, in some situations, the need to separate patients so quickly made it difficult to classify them accurately… (10).

…During triage, it was hard to assess patients and determine the treatment order, as many arrived at the tent simultaneously… (4).

#### 3.2.5 Lack of Personal Hygiene

Participants reported that the lack of basic hygiene facilities hindered their personal cleanliness during the disaster response.

…there were no toilets, no bathrooms, no showers, nothing. After we left for the first week, we saw that they were gradually established… (1).

…from the day we left, for the first three days I was not able to wash my hands. I never saw a running tap. For the first three days we could hardly touch soap and water. We had disinfectants that we used in hospitals… I didn't go to bathe for 15 days. I didn't bathe and had to cut my hair… (9).

#### 3.2.6 Nurses' Lack of Knowledge and Skills

Nurses reported that the lack of information reduced the effectiveness of their disaster response, although they volunteered to help effectively when disasters occurred.

…many of us did not have enough knowledge… For example, one of our friends who went came back when he saw the conditions there. Disaster training should be strengthened. It should not just be on paper. They should really show us what they expect from us in such situations, so that we can go in a conscious way and not get in the way… (11).

…The lack of training is a major issue. We didn't receive any disaster management training, and not knowing what to do during such events was extremely challenging… (20).

### 3.3 Main Theme 3: Psychosocial Challenges

The results obtained under the heading ‘psychosocial challenges’ were grouped into four sub‐themes: Emotional burden, feeling of inadequacy, safety concerns, challenges of post‐disaster recovery.

#### 3.3.1 Emotional Burden

Participants reported that the traumas they experienced during the disaster, combined with their interactions with *earthquake victims*, created a significant emotional burden, leaving lasting psychological effects.

…Constant interaction with the earthquake victims led to the formation of emotional bonds. However, these bonds became a psychological and emotional burden. Seeing their pain, listening to them was overwhelming for me. While empathising with them, I sometimes lost myself… (20).

…What I saw affected me deeply. The bodies, the collapsed buildings… Seeing so much trauma every day made it difficult to carry on as if nothing had happened…. (14).

…Passing by the bodies in the hospital and having to number them was very difficult. You don't feel anything in the moment, but thinking about it afterward makes it even harder… (20).

#### 3.3.2 Feeling of Inadequacy

Participants expressed feelings of helplessness and inadequacy in the face of the destruction they encountered during and after the disaster. They noted that the limitations of what could be done during the response to the disaster affected them deeply.

…When I went to the city, I felt helpless in the face of what I saw and there was a feeling of not being enough. We were not enough for anything. We saved as many people as we could… (4).

…Sometimes I felt inadequate because I was trying to do so much, and it felt like I wouldn't be able to handle it all… (13).

#### 3.3.3 Safety Concerns

Participants expressed concerns about their safety due to aftershocks and the risk of structural collapse following the disaster. They mentioned that maintaining a constant state of high alert was both physically and psychologically draining.

…There were aftershocks… One of the walls of the hospital had collapsed and frankly I was worried about what to do if another earthquake… (6).

…Aftershocks were constant… being on high alert all the time was psychologically exhausting… (20).

#### 3.3.4 Challenges of Post‐Disaster Recovery

Participants highlighted the psychological and social difficulties they experienced in returning to normal life after the disaster. They stated that the intense experiences in the disaster zone made it difficult to resume everyday life, and emotional distress, long‐term effects, and lasting memories made recovery even more difficult.

…When we came back, I felt like I had ‘forgotten’ normal life. I had such an intense experience that everything here seems so simple and ordinary… (14).

…Coming back after the disaster and expecting everything to go back to normal was very difficult. Many of my friends and I felt a huge emotional void when we returned… (15).

…It was hard to start again. After the disaster, nothing was the same, and I didn't know how to cope with those traumas. Even though years have passed, some situations still feel fresh to me… (13).

…I'm afraid even to talk about it. We live in Istanbul, we have seen what can happen to us and believe me, I still feel like an earthquake at night… It happened two days ago… I suddenly woke up and said ‘an earthquake is happening’… I woke up my husband… and I had this experience very often after my return from Maraş… (17).

…the trauma we witnessed is something that can affect us for years. I find myself constantly remembering those moments… (20).

### 3.4 Main Theme 4: Positive Experiences

In analyzing the findings under the heading of ‘positive experiences’, they were grouped into two sub‐themes: ‘professional satisfaction’, ‘team support’.

#### 3.4.1 Professional Satisfaction

Participants expressed that their experience in the disaster area had been professionally satisfying and emphasized in particular the value of their assistance in making a difference in people's lives, where even a small contribution had great significance.

…the way the people there looked at us, their respectful behaviour towards us, after seeing them, I felt really proud to have chosen this profession… (11).

…When I was working in the earthquake zone, a child brought me a piece of cake. Seeing that we were tired, he said, ‘Take this’. This small but meaningful gesture gave me immense professional satisfaction. Being able to touch even one life is incredibly valuable…. (9).

…To be able to help a person rescued from the rubble, even just lifting a stone, was enough for me… (14).

…When people are happy, you feel you've achieved something. Now, as a nurse, I feel I've touched many lives… (8).

…I gained a lot from working in the disaster area. Being able to touch even one little life was incredibly valuable. I have no regrets; it was a very valuable experience… (5).

#### 3.4.2 Team Support

Nurses emphasized that the support and motivation within the team played a crucial role during this challenging period, noting that they were able to overcome difficulties by continuously boosting each other's morale.

…I tried to motivate my team. I kept telling my friends, ‘We can do it, we're here because we came to help’ (13).

…Sometimes, by supporting each other, we tried to stay strong in these difficult conditions. As a team, we motivated each other (15).

…Supporting each other not only helped us stay on task but also made us stronger psychologically (14).

## 4 Discussion

The demand for health services increases after a disaster, and nurses play a critical role in disaster response. Identifying the experiences of nurses in the disaster process can be a guide for disaster preparedness. The nurses' views on the main theme and sub‐themes were discussed with the findings of other studies.

Nurses stated that their involvement in disaster management processes was voluntary or non‐voluntary in the interviews. The results of our study are in parallel with the literature. The study by Çetinkaya Özdemir et al. (2023) and Abdi et al. (2021) found that nurses from different cities came to the earthquake area with their own means and volunteered in search and rescue teams where hospitals, tents, ambulances, and coordination were lacking. In the study by Doğan et al. (2024), nurses stated that they voluntarily continued their duties despite their concerns about the safety of the hospital building, as well as the happiness of being able to help earthquake victims in the acute period after the earthquake, and that this situation increased their professional satisfaction. Regardless of the difficulties and conditions, nurses tried to fulfill their basic roles and obligations as caregivers continuously and selflessly. This finding shows the commitment and sacrifice of nurses to their professional obligations in disaster processes and also shows that the importance of nurses in disasters is an undeniable fact.

Participants reported that non‐voluntary assignments were made after the voluntary lists had been finalized. Some participants noted that these non‐voluntary assignments posed additional challenges, especially for individuals directly affected by the disasters. Similarly, in the study by Doğan et al. (2024), nurses living in the disaster area defined nursing as a duty after the earthquake and stated that they wanted to be with their families and loved ones affected by the earthquake, but they had to leave them and come to the hospital because they were called to duty. That nurses who are concerned about the safety of their families and loved ones perceive their duty as an obligation is an expected finding. This finding points to the need for more detailed research into the role and impact of voluntary and non‐voluntary deployment in responding to disasters.

The “practice challenges” theme highlights the inevitable difficulties nurses face during disasters due to the unpredictable and complex nature of such events. The findings of this study are supported by the literature. Studies have highlighted that nurses experience challenges such as lack of coordination, inadequate institutional support, and ineffective management during disasters (Abdi et al. 2021; Farokhzadian et al. 2024; Scrymgeour et al. 2020). Additionally, shortages of medical supplies and equipment create problems in patient care and treatment (Farokhzadian et al. 2024; Altuntaş et al. 2023; Doğan et al. 2024). Deficiencies in triage practices have been reported to lead to inappropriate patient classification, delays in treatment, and ethical dilemmas (Gustavsson et al. 2022; Kivi et al. 2023; Abdi et al. 2021). Furthermore, the challenges and limitations of providing healthcare in emergency conditions, such as in makeshift tents, have also been highlighted (Tunç 2023). Consistent with the findings of this study, the literature highlights that limited access to personal hygiene facilities following disasters poses a major challenge in maintaining health and preventing further harm (Altuntaş et al. 2023; Dereli and Yıldırım 2023; Salik et al. 2024). Finally, in line with the literature, nurses have reported inadequate knowledge and skills in disaster management, which negatively impact their ability to respond effectively, and they have emphasized the insufficiency of disaster education (Altuntaş et al. 2023; Rizqillah and Suna 2018). Researchers suggest that the neglect of disaster training for nurses is not limited to a specific country but constitutes a global concern, emphasizing that standardized and continuous disaster training plays a significant role in addressing these gaps (Altuntaş et al. 2023; Taşkıran and Baykal 2019). These findings highlight the urgent need for improved planning, coordination, training, resources, and infrastructure in disaster management. They also underscore the importance of implementing strategic measures to enable nurses to play a more effective role in disasters.

In this study, nurses were observed to experience psychosocial challenges, including emotional burden, feelings of inadequacy, safety concerns, and difficulties in post‐disaster recovery. Various studies have shown that nurses experience emotional exhaustion, and most of them are in need of psychological support (Abdi et al. 2021; Farokhzadian et al. 2024; Altuntaş et al. 2023; Doğan et al. 2024; Çetinkaya Özdemir et al. 2023). It was emphasized that anxiety, burnout, emotional burden, depressive symptoms, and secondary traumatic stress are the most common psychological reactions of nurses working with earthquake victims (Salik et al. 2024; Doğan et al. 2024; Çetinkaya Özdemir et al. 2023). Bektaş Akpınar and Aşkın Ceran's (2020) study found that nurses' inadequacies in treating earthquake victims, carrying the emotional burden of earthquake victims, and meeting these patients' care needs led to burnout. Furthermore, another study in China observed that nurses interviewed 5 years after the disaster cried while describing their earthquake experiences and were still very emotional (Li et al. 2015). These results highlight the importance of psychologically empowering nurses and increasing their psychological resilience by providing psychological support in the disaster area in order to prevent psycho‐emotional injury among nurses in the disaster work environment.

The findings of this study show that nurses who responded to disasters experienced significant positive aspects, categorized into two sub‐themes: professional satisfaction and team support. These findings are consistent with the existing literature, which highlights the rewarding aspects of disaster nursing despite its inherent challenges (Lee et al. 2017; Chen et al. 2020; Doğan et al. 2024). In the study by Doğan et al. (2024), some nurses described the nursing profession as “satisfying” after the earthquake. Similarly, in the study by Şermet Kaya and Gülnur Erdoğan (2024), nurses reported great satisfaction in their profession due to the physical and emotional impact they had on others and recognized its true value. Additionally, the study emphasized the central role of helping others and fostering a sense of teamwork throughout the process. These positive experiences underscore the intrinsic value of disaster care and highlight the importance of promoting supportive team dynamics to increase resilience and satisfaction among healthcare professionals.

## 5 Conclusion

The results of this study highlight the various physical, psychosocial, and organizational challenges nurses face during disasters, and the fact that nurses reach and care for patients in adverse physical conditions and with limited resources. The issues discussed under the theme of practical challenges have a significant impact on the coordination and effectiveness of disaster response teams, thereby severely limiting the quality and accessibility of post‐disaster health services. The theme of psychosocial challenges highlights the emotional and psychological impact of the disaster on nurses, including emotional distress from interacting with earthquake victims, feelings of inadequacy in the face of overwhelming destruction, constant safety concerns due to aftershocks, and difficulties in adjusting to normal life due to lingering trauma. On the other hand, the positive experiences theme highlights the professional fulfillment nurses gained from their impactful contributions and the strength they drew from team support during the challenging disaster response.

### 5.1 Implications for Policy and Practice

The findings highlight the need to improve disaster preparedness and response strategies to better support nurses during crises and improve healthcare delivery. Clear crisis management protocols and robust communication systems are essential for effective coordination. Standardized disaster training programs should be integrated into nursing education, providing nurses not only with theoretical knowledge but also with practical experience in adapting to disaster conditions, and these programs should be regularly updated.

Healthcare organizations should include strategic measures in their disaster plans to secure resources such as medical supplies, equipment, and medicines, and to manage resources effectively. The safety and accessibility of the physical environment are critical, so appropriate working conditions for disaster response teams should be ensured, and the design of temporary health facilities should be improved. Psychosocial support systems should be developed to alleviate the psychological burden of nurses working in disaster areas. Finally, promoting a culture of teamwork and mutual support will help nurses cope with stress and provide moral support.

In addition, more research should be conducted on the roles, experiences, and difficulties faced by nurses in disaster situations. Such studies can form the basis for better management of disaster health services and contribute to the development of future disaster response strategies.

### 5.2 Limitations

This study highlights the unique experiences of nurses in Türkiye. However, the findings cannot be generalized to all cultures and contexts. Disaster preparedness experiences are influenced by cultural, economic, social, and educational factors. Therefore, it is recommended that similar studies be conducted in other societies to gain a broader understanding of nurses' disaster preparedness experiences. The sample size, although adequate for qualitative saturation, may limit the generalizability of the findings. Additionally, similarities in some demographic characteristics may have influenced the diversity of perspectives, which is a consideration for future studies.

## Author Contributions

**Cafer Açık:** conceptualization, data curation, methodology, writing – original draft, investigation. **Tuğba Yeşilyurt Sevim :** conceptualization, writing – review and editing, supervision, methodology, writing – original draft, investigation.

## Ethics Statement

This study was conducted according to the guidelines of the Declaration of Helsinki, and all procedures involving human subjects were approved by the İstinye University Human Research Ethics Committee (Decision number and date: 23‐243/September 22, 2023).

## Conflicts of Interest

The authors declare no conflicts of interest.

## Data Availability

The data that support the findings of this study are available from the corresponding author upon reasonable request.
